# Acknowledgement to Reviewers of *Epigenomes* in 2018

**DOI:** 10.3390/epigenomes3010002

**Published:** 2019-01-11

**Authors:** Epigenomes Editorial Office

**Affiliations:** MDPI, St. Alban-Anlage 66, 4052 Basel, Switzerland

Rigorous peer-review is the corner-stone of high-quality academic publishing. The editorial team greatly appreciates the reviewers who contributed their knowledge and expertise to the journal’s editorial process over the past 12 months. In 2018, a total of 21 papers were published in the journal, with a median time to first decision of 15 days and a median time to publication of 38 days. The editors would like to express their sincere gratitude to the following reviewers for their cooperation and dedication in 2018:
Bagnat, MichelKumar, ManuBakht, MartinLewsey, Mathew G.Cava, ClaudiaLiss, Andrew S.Chen, Pao-YangLomberk, GwenCheng, Yuen YeeLunardon, AliceChüeh, Anderly C.Maleszka, RyszardCorces, VictorMione, MarinaCrisp, PeterMoin, MazaharDe La Serna, IvanaMuchardt, ChristianDi Pizio, AntonellaNandi, SaikatDoni Jayavelu, NareshNestor, Colm E.Elliott, HannahOlova, NellyEl-Rifai, WaelPérez-Lluch, SílviaFu, BeidePerucho, ManuelFuso, AndreaRoccaro, AldoGao, LongRönn, TinaGood, Deborah J.Satyaki, P.R.V.Gradziel, Thomas M.Saze, HidetoshiGuan, XueyingSchweiger, Michal R.Harris, R. AlanShen, Che-Kun JamesHassan, QuamarulToh, Tan BoonHernandez-Vargas, HectorTrollope, AlexandraHo, Eric S.Tucci, ValterHuang, JijunUlicna, LiviaIlmer, MatthiasVan Ness, BrianIm, Chang-NimVarotto, SerenaJha, SudhakarVélez-Cruz, RenierJHANWAR, SHALUVerde, GaetanoJin, PengWang, Bang-AnJung, Chol-HeeZaidi, Syed Shan-e-AliKandimalla, RajuZapico, Sara C.Kast, Richard EricZhu, JianKleeff, JörgZhu, Jian-KangKnapp, StefanZhu, Qian-HaoKramer, Jamie M.


